# Revisiting the speaker discriminatory power of vowel formant frequencies under a likelihood ratio-based paradigm: The case of mismatched speaking styles

**DOI:** 10.1371/journal.pone.0311363

**Published:** 2024-12-10

**Authors:** Julio Cesar Cavalcanti, Anders Eriksson, Plinio A. Barbosa, Sandra Madureira

**Affiliations:** 1 Department of Linguistics, Stockholm University, Stockholm, Sweden; 2 Applied Linguistics and Language Studies Graduate Program, Pontifical Catholic University of São Paulo, São Paulo, Brazil; 3 Institute of Language Studies,University of Campinas, Campinas, Brazil; University of Aizu, JAPAN

## Abstract

Differentiating subjects through the comparison of their recorded speech is a common endeavor in speaker characterization. When using an acoustic-based approach, this task typically involves scrutinizing specific acoustic parameters and assessing their discriminatory capacity. This experimental study aimed to evaluate the speaker discriminatory power of vowel formants—resonance peaks in the vocal tract—in two different speaking styles: *Dialogue* and *Interview*. Different testing procedures were applied, specifically metrics compatible with the likelihood ratio paradigm. Only high-quality recordings were analyzed in this study. The participants were 20 male Brazilian Portuguese (BP) speakers from the same dialectal area. Two speaker-discriminatory power estimates were examined through Multivariate Kernel Density analysis: Log cost-likelihood ratios (*C*_*llr*_) and equal error rates (EER). As expected, the discriminatory performance was stronger for style-matched analyses than for mismatched-style analyses. In order of relevance, F3, F4, and F1 performed the best in style-matched comparisons, as suggested by lower *C*_*llr*_ and EER values. F2 performed the worst intra-style in both *Dialogue* and *Interview*. The discriminatory power of all individual formants (F1-F4) appeared to be affected in the mismatched condition, demonstrating that discriminatory power is sensitive to style-driven changes in speech production. The combination of higher formants ‘F3 + F4’ outperformed the combination of lower formants ‘F1 + F2’. However, in mismatched-style analyses, the magnitude of improvement in *C*_*llr*_ and EER scores increased as more formants were incorporated into the model. The best discriminatory performance was achieved when most formants were combined. Applying multivariate analysis not only reduced average *C*_*llr*_ and EER scores but also influenced the overall probability distribution, shifting the probability density distribution towards lower *C*_*llr*_ and EER values. In general, front and central vowels were found more speaker discriminatory than back vowels as far as the ‘F1 + F2’ relation was concerned.

## Introduction

The comparison of speakers through the analysis of their speech and voice records is a common practice in speaker characterization with implications for forensics. The primary aim of such comparisons is generally to assess the probability that sets of speech samples originate from the same source (same speaker) or different sources (different speakers). In an acoustic-oriented approach, this process involves a meticulous examination of an array of acoustic-phonetic parameters, including melodic, spectral, and temporal parameters [1, 2].

In a previous experimental study [3], we investigated the speaker discriminatory power of individual vowel formants within a single speaking style, specifically spontaneous dialogues conducted through mobile phone conversations. The statistical analyses employed were primarily based on a frequentist inferential statistical approach. In this follow-up study, we address the same issue using different testing procedures aligned with recent trends in forensic sciences: a likelihood ratio-based statistical paradigm. Understanding the consistency of outcomes when applying different statistical methodologies is therefore of critical importance. Additionally, our focus has shifted from analyzing identical twin speakers to examining non-twin comparisons only. This decision stems from the fact that non-twin comparisons are more representative of real-world speaker comparison scenarios, whereas comparisons involving highly similar individuals, such as identical twins, are relatively rare.

### Vowel formant frequencies

Regarding the array of parameters commonly assessed in forensic speaker comparison (FSC) practice, formant frequencies are among the most common [1]. Previous experiments also suggest that, when dealing with high-quality recordings, formant frequencies are among the most speaker-discriminatory parameters when compared to melodic and timing estimates [2].

In general, the FSC task is commonly carried out considering F1, F2, and F3 due to the inaccessibility imposed by the telephone bandwidth to higher frequencies. However, recent technological advances in online messaging communication (e.g., WhatsApp and Telegram) have widened the possibilities of assessing higher frequencies in evidence materials [4].

Regarding speech production, vowel segments, like other speech sounds, are assumed to convey information in three dimensions: linguistic, social, and idiosyncratic, as mentioned by [5]. These dimensions are the driving forces of variation in speech production.

Regarding acoustic vowel specification, the quality of a vocalic segment (e.g., /i/, /u/, or /a/) is primarily correlated to the frequency of the first and second speech formants [6], namely F1 and F2, produced by the proper manipulation of mouth opening and constriction location. Variations in these dimensions are related to the degree of articulatory precision required for producing a given vowel [7]. Furthermore, higher formants such as F3 (i.e., somewhat related to vowel configuration, as in the case of front rounded vowels) and F4 are commonly referred to as being more speaker-specific, conveying more speaker-discriminant information [4]. These formants are also seen as related to voice quality aspects in spoken and singing voice [8].

As mentioned by [9], the position of the higher formants in the spectrum, such as F3 and F4, is largely determined by the vocal tract characteristics. Moreover, in the experiment conducted by [7], the authors observed that while F2 tended to increase in frequency as the point of constriction moved forward from the glottis, there was only a small increase in F3 as the mouth opening increased in size and became less rounded during the referred movement. The rate of increase in F3 depended mostly on the constriction size.

Studies suggest that the laryngeal cavity (LC) has its unique acoustic properties and is independent of the rest of the vocal tract [10, 11], showing that the elimination of the LC results in the suppression of F4 while other formants remain unaffected [11]. Furthermore, experiments using acoustic models have demonstrated that changes in the shape of the LC can have a significant impact on F4. However, it should be acknowledged that those experiments were carried out in an experimental setting using a particular artificial vocal tract, which limits the possibility of generalizations to real vocal tracts.

Some common sources of inter-speaker variability concerning vowel configuration are reported in the literature, including differences as a function of sex, dialect, age, speaking rate, and speaking style [12–15]. For the present study, we will focus on the latter variable: speaking style, mainly on its impact on the speaker-discriminatory power of formants.

### Speaking style

Some studies have explored the levels of speaker variability concerning vowel space configuration as a function of different speaking tasks. Among these, some attention has been paid to the effects of slow and fast speech [14, 16], “clear speech” [17, 18], the nature of the spoken material– whether scripted or not [15], and “loud speech”. There are, of course, a plethora of other factors associated with changes in speaking style; however, we will focus on those that are pertinent to our study.

The study conducted by [14] aimed to examine the size of the acoustic vowel space in talkers identified as having slow and fast habitual speaking rates. Thirty participants, 15 slow and 15 fast talkers aged 18 to 35 years, were selected for the experiment. All speakers had dialects from the upper Midwest of the USA. Participants were asked to read a text at habitual and maximal speaking rates. Based on the total reading time and overall speaking rate of each participant’s performance, talkers were classified into slow and fast groups.

The results indicated no difference in the average size of the vowel space between slow and fast talkers and no relationship across talkers between vowel duration and formant frequencies. One observed difference between the slow and fast talkers was in the inter-talker variability of the vowel spaces, which was greater for the slow talkers of both sexes. However, the hypothesis of a cross-speaker, rate-dependent size of the vowel space was not supported by the data, as the average vowel spaces of the two groups of speakers were virtually identical, as were the average formant frequencies for the individual vowels.

“Clear speech” defined as “a speaking style that is used by talkers when they know they may have difficulty being understood” [19], which is typically slower than conversational speech [20], has also been studied. In a comprehensive study conducted by [18], it was observed that across all 41 talkers, the first formant (F1) increased significantly in clear speech for 7 out of the 10 English vowels assessed. The predominant trend in the F1 data showed an overall increase, which suggests that speakers typically lower their jaw and tongue more in clear speech, selectively enhancing the acoustic distance between high and low vowels. For the second formant (F2), the direction of change varied depending on the vowel category. Specifically, front vowels exhibited significantly higher F2 values, while back vowels displayed lower F2 values in clear speech. This combination of increased F2 for front vowels and decreased F2 for back vowels resulted in an expanded F2 range.

In [15], F1 and F2 formants of the French vowels /i/, /a/, and /u/ were assessed in six speech tasks: (i) describing “bizarre details” in a picture; (ii) providing a more comprehensive picture description with a narration style; (iii) same as in situation (ii), but the speaker was asked through a headset to provide other complementary information in the picture; (iv) the speaker was told that a new person who had never seen the picture would have to reproduce it based on the information he/she delivers; (v) the speaker engages in a spontaneous conversation with an experimenter; (vi) the speaker was asked to read a word list presented in random order.

The results revealed that the four tasks where the speaker had to describe a picture shared common characteristics. The spontaneous speech task appeared different from the other situations. In general, tasks in which the speaker had to interact frequently with an interlocutor tended to show substantial contrast with non-interactional tasks. As suggested by topological observations of the vowels in the bi-dimensional acoustic plane, there was a clear tendency for more centralized formant values in spontaneous speech compared to the other tasks (i.e., picture descriptions).

Experiments have also been conducted to study how the acoustic configurations of vowel formant frequencies are affected by “loud speech”. In the study carried out by [21] with eleven German-speaking female speakers, the subjects were instructed to produce normal and loud speech in 3 tasks: i. reading sentences with the target vowels /i: a: u:/, ii. answering questions that included target words with controlled consonantal contexts with varying vowel types and a text recall task. Loudness variation was elicited naturalistically by changing interlocutor distance. F1 and F2 formant frequencies were obtained from the stressed vowels in the targets.

As reported by [21], comparisons across vowels indicated that high, tense vowels showed limited formant variation as a function of loudness. Analysis of /i:, a:, u:/ across speech tasks revealed a reduction in vowel space during the retelling task compared to the other tasks (i.e., reading and answering specific questions). Loudness changes for F1 were consistent in direction but variable in extent, with few significant results for high, tense vowels. Moreover, results for F2 were quite varied and seldom significant. Overall, the authors observed that speakers differed in how loudness and task affected formant values, suggesting that loud speech does not always lead to changes in vowel formant patterns.

A crucial aspect, particularly concerning speaker characterization with implications for forensics, involves the mobile phone speaking condition and its possible effects on acoustic-phonetic parameters. Studies such as [22] suggest that speaking on a mobile phone constitutes a unique speaking style due to the specific characteristics of this condition and the significant changes it imposes on acoustic-phonetic descriptors. However, most available studies on speaker discrimination have primarily focused on the artificial effects of telephone/mobile phone bandwidth transmission, which may represent a limitation. This focus makes it challenging to isolate the specific impact of speaking style alone from the artificial changes imposed by the transmission channel.

Among some studies involving mobile phone-transmitted speech, we highlight the one conducted by [23] on the effects of mobile phone transmission on vowel formant frequency measurement. In that study, six male and six female English speakers (with average ages of 23 and 24, respectively) read a short passage while speaking through a mobile phone. Two simultaneous recordings were made and compared: one at the far end of the phone line (mobile phone-transmitted) and the other via a microphone directly in front of the speaker (direct recording). Measurements of F1, F2, and F3 were taken from stressed vowels in both recordings.

Results from that study suggested that, due to the filtering effect of the phone transmission, measured F1 frequencies for most vowels were higher than their counterparts in the direct recordings. On average, measured F1 values in the mobile phone condition were 29 percent higher than in the direct condition. Furthermore, F2 measurements were not significantly affected. Measured F3 frequencies were also generally unaffected by mobile phone transmission. However, some speaker-related exceptions were found, particularly for subjects with relatively high F3s. In these cases, the mobile phone recordings tended to yield significantly lower values.

Regarding Brazilian Portuguese, the study by [24] focused on determining the extent of acoustic-phonetic changes caused by mobile phone band-pass filters and transmission effects on oral vowels. Recordings of 10 male speakers were analyzed in two conditions: mobile phone-transmitted and direct speech. The analyses showed that mobile phone band-pass filtering shifted vowels with low F2 values upwards and vowels with high F2 values downwards. Furthermore, it was verified that telephone transmission affected speakers differently. The analysis of the effects on the vowel space demonstrated that the increase in measured F1 values in the mobile phone situation resulted in a global downward displacement of the vowel space. Additionally, the decrease in measured F2 values for front vowels and the increase for back vowels tended to reduce the area of the vowel space for most subjects.

In light of previous research, it is reasonable to ask: what is the impact of speaking style alone on the discriminatory power of vowel formant frequencies when removing the artificial changes imposed by the transmission channel? This study aims to address that question.

In contrast to [3], the present study also explores the combined discriminatory power of different formants. Furthermore, a different speaking style (*Interview*) was included in the analyses, allowing us to compare findings obtained from spontaneous *dialogue* with those from the *interview* style. Comparing the same subjects across two different speaking styles enables the assessment of formant discriminatory power when mismatched speech materials are confronted. Furthermore, the impact of data sampling on the estimation of discriminatory power metrics has also been considered, as described in the methods section.

### Some forensic implications

Theoretical and methodological standards have been carefully examined and recommended to determine the most effective parameters for applications in speaker comparison. A set of criteria proposed by [25] encompasses the following desirable attributes that an acoustic measure should exhibit: a large degree of inter-speaker variability, minimal intra-speaker variability, robustness against attempts at disguise or imitation, robustness against the effects of transmission channels, frequent occurrence (*availability*), and ease of measurement (*measurability*).

Concerning the first two criteria (intra- and inter-speaker variation), there is a notable lack of studies in Brazilian Portuguese that examine the extent of variation in phonetic-acoustic parameters across different speaking styles, particularly those commonly encountered in forensic contexts, such as spontaneous dialogues and interviews.

A recurring practice in forensic speaker analysis is the comparison of speech materials from the same subject in at least two different speaking styles: (i) establishing a spontaneous dialogue with a familiar interlocutor, often resulting from a telephone interception, called “questioned” speech material; and (ii) being subjected to an interrogation (i.e., interview) with an unfamiliar interlocutor, resulting in a speech sample called “reference” material. The process of comparing such materials collected under different conditions is accompanied by a reduction in the dimensionality and representativeness of the samples [6].

As highlighted in [6], assessing differences between samples becomes a major challenge as there is generally no control over the situations in which data are collected. In most cases, the expert is led to confront a reference recording collected in a context that is almost or entirely different from the context of the standard sample acquisition due to the presence and/or absence of condition-specific variables. Therefore, in this experiment, we aim to isolate and assess one such condition-specific variable: speaking style.

## Materials and methods

The present study, registered under protocol 95127418.7.0000.8142, was evaluated and approved by the ethical committee at Campinas State University (UNICAMP). All participants voluntarily agreed to be part of the research verbally and by signing a participant consent form. All personal information regarding the participants is kept private.

### Participants

The participants are 20 subjects, namely 10 identical twin pairs, all male, Brazilian Portuguese (BP) speakers from the same dialectal area (state of Alagoas). The participants’ ages ranged between 19 and 35 years, with a mean of 26.4 years.

Although the participants in this study were originally part of a twin study project that analyzed potential acoustic-phonetic differences in the speech productions of identical twin pairs, this study will focus on comparing non-twin subjects. For more information on the experiments involving twin speakers, the reader is referred to [3, 26–28].

The inclusion and exclusion criteria established for the referred original project were the following. Inclusion criteria: i. Identical twins; ii. male speakers; iii. same dialect; iv. aged between 18–45 years; v. with at least elementary school completed (High School). Exclusion criteria: i. Reported hearing loss or speech disorder, ii. identical twins raised apart; iii. identical twins that lived apart from each other for more than five years.

#### Speaker groups

All identical twin pairs were codified with letters and numbers, such as A1, A2, B1, B2, C1, C2, D1, D2, and so on. The same letters indicate that the speakers are identical twins and, therefore, related. Unlike [3], this study focuses solely on non-twin comparisons. To achieve this, the 20 participants were divided into groups of 10 speakers, each comprising non-twin speakers only. There are many ways to form groups of 10 speakers out of 20, avoiding the inclusion of twin pairs. The number of groups should not be too small, to prevent group-specific patterns from heavily impacting the outcomes, and not too large, to ensure computational feasibility. To strike this balance, we decided to work with 150 random speaker groups.

### Recordings

The recordings were carried out in silent rooms in the cities where the participants resided. All recordings were carried out with a sample rate of 44.1 kHz and 16-bit amplitude resolution, using an external audio card (Focusrite Scarlett 2i2) and two headset condenser microphones (DPA 4066-B).

The speech material analyzed regards: i. spontaneous telephone conversations (*dialogues*) between familiar speakers (twin pairs); and ii. *interviews* between the participants and the experimenter (the first author). The recordings were carried out in two different contemporaneous sessions (i.e., in the same day). The specificities regarding each speech material and the context for their elicitation are presented in the following.

#### Recording session I

During the first recording session, the subjects were placed in separate rooms where they could not see, hear, or interact with each other directly. Using mobile phones, the speakers were instructed to initiate a conversation while being recorded simultaneously by high-quality professional headset microphones. The participants had the freedom to choose the topics of their conversations.

An important aspect of the experimental design used here is the high level of control over the variable of “familiarity” between the speakers. In forensic contexts, speakers are typically familiar with each other, which can affect their speech and level of self-monitoring. The recording method used in this stage of the experiment aimed to elicit speaking adaptations that are typical of mobile phone speaking conditions. The ultimate goal was to approximate the experimental conditions to those encountered in more realistic scenarios, as done in previous studies [3, 26–29].

#### Recording session II

During the second recording session, the experimenter (the first author) interviewed each speaker. The participants were asked to describe their daily routine, starting from when they usually wake up until they go to bed, as well as what they typically do during their free time. After describing their current routine, the participants were asked to reflect on their routines from the previous week, month, and year, and to discuss any changes that occurred over the past year.

Apart from the different speaking styles elicited, an important aspect of this recording stage is the intentional reduction in familiarity between the interlocutors. This was done to simulate a setting where individuals are interrogated by an unfamiliar interviewer.

### Data transcription and extraction

The data segmentation and transcription were performed manually using the Praat software [30]. All data were transcribed and reviewed by the first author, who is a phonetician, a certified speech-language pathologist, and a native speaker of the dialect studied. Speech portions pertinent to the present analysis were divided into three different tiers in the Praat TextGrid, as follows:

1. **Dialogue part:** different portions/parts of the dialogues throughout the recordings, e.g., beginning, middle, and final parts;
2. **Speech chunks:** speech intervals, in most cases corresponding to inter-pause intervals (i.e., stretches of speech between long silent or filled pauses);
3. **Oral monophthongs:** oral monophthongs produced within speech chunks;

The focus of the present research was the seven phonemic vowels of BP /i, e, ε, a, ͻ, o, u/. We refer to these vowels as possessing different qualities, namely, different perceptual and acoustic characteristics to them. Hence, the term ‘vowel quality’ is used. Regarding the number of tokens for each vowel in both speaking styles, these were as follows (from higher to lower): 5,995 /a/, 2,910 /i/, 1.838 /u/, 1,684 / ε/, 1,479 /e/, 1,063 /o/, 973 /ͻ/, resulting in a total of 15,942 tokens.

Vowels were manually segmented and transcribed based on auditory and acoustic criteria, specifically by carefully listening to the speech segments and observing the appearance and disappearance of F2 energy in the broad-band spectrogram. F1-F4 values were automatically extracted from the midpoint of the labeled vowels using a Linear Predictive Coding (LPC) technique. The maximum frequency range for formant extraction was set to 5000 Hz, identifying five formants using an LPC order of 10. Possible measurement errors were subsequently checked using the Interquartile Range to detect outliers within speakers, styles, and vowels of the same speaker.

The parameter extraction was performed using a Praat script cf. [31] developed by the third author. The script generates a text file containing speaker identity, vowel labels, vowel duration in seconds, and formant frequencies in Hertz. Statistical analyses were conducted using formant frequency measurements in Hertz.

### Statistical analysis

All statistical analyses were carried out in the R environment [32]. The main justification for focusing on comparisons among non-twins is that such data can be considered more realistic from a general standpoint. In everyday life, individuals may share similarities in aspects such as sex, age, dialect, and education level, but the extreme similarities seen in twin pairs are relatively rare.

The order of the subsections presented below reflects the steps taken during the statistical analysis process.

#### Down-sampling procedure

Considering the intricate nature of spontaneous speech, characterized by variations in lexical items, phrase length, syllable structures, and different amounts of speech material produced by the subjects, an n-size compensation was performed using a down-sampling procedure, as described below.

The procedure consisted of randomly sampling vowel data points so that all classes (here, speakers) have the same frequency as the minority class (i.e., the speaker with the fewest number of vowel data points, i.e., 208 in *interview*, and 397 in *dialogue*). As a result, all individuals will present the same number of vowels, yielding an n-size balanced data set. The primary justification for employing such a procedure lies in the necessity of reducing the discrepancy concerning the number of observations across parameters and speakers. This does not imply that the seven vowels were equally distributed after downsampling, as that would be unrealistic. For example, the most extreme vowels /a/, /i/, and /u/ are typically more frequent, and therefore more likely to be overrepresented in the downsampled data. We chose to maintain such a distribution, as it may better reflect real-world conditions.

Since data sampling is a random process, such a procedure was repeated 100 times, allowing the computation of cumulative speaker discriminatory power metrics, being less susceptible to the effects of a selection bias, as in [2, 33]. Every time the data was re-sampled for all speakers up to 100 times (except for that one speaker with the fewest number of vowel data points), a new data set configuration was obtained. After that, speaker discriminatory power statistical metrics were computed, as described further.

Multiple downsamplings were conducted during mismatched speaking style comparisons to maintain consistency in the number of observations for each speaker and speaking style during comparisons across various speaking styles. The downsampling procedure outlined here occurs before the computation of LR-like scores and is reiterated whenever a new calculation takes place.

A total of 9,384 tokens were available for *dialogue* and 6,558 tokens for *interview*, resulting in a sum of 15,942 vowel tokens. Outliers were automatically checked within the speaker, vowel, and speaking style using the less conservative interquartile range (IQR) by 2.5 to avoid the possible exclusion of biometric information in the data. After outlier removal, we arrived at 15,459 vowels (9,103 for *dialogue* and 6,356 for *interview*). Post downsampling, taking into account speakers with the fewest number of vowel data points (382 for *dialogue* and 202 for *interview*), 7,640 vowels were tested per iteration for *dialogue* and 4,040 vowels for *interview* (11,680 in total).

#### Likelihood ratios (LR)

The computation of Likelihood Ratios (LR) can be understood through the generic representation given by Formula 1, where LR is the likelihood ratio; *E* represents the evidence, i.e., the measured difference between the samples of known and questioned origin; *p*(*E* ∣ *H*) is *the probability of E given H*; respectively *H*_*s*_ is the same-speaker hypothesis, and *H*_*d*_ is the different-speaker hypothesis, i.e., same-origin and different-origin hypotheses [34].

$$\begin{array}{c} LR=\frac{p(E|H_{S})}{p(E|H_{d})} \end{array}$$ (1)

For instance, when comparing samples based on mean F1 values for the vowel /a/, the evidence (*E*) would be the observed mean F1 values for /a/ vowels from the speakers being compared. The LR-like score would then be calculated by comparing the distribution of observed mean F1 values under the hypothesis that they come from the same speaker (*H*_*s*_) versus the distribution under the hypothesis that they come from different speakers (*H*_*d*_).

LR-like scores necessitate individual calculations for formants of different vowels. For example, when evaluating samples based on parameters like mean F1 and F2, a separate LR-like score must be computed for each vowel under consideration based on the desired combination of formants. In a univariate scenario, such as F1 values for /a/ vowels, the comparison involves assessing absolute differences in F1 values within /a/ vowels from the same speaker (*similarity*) versus those from different speakers (*typicality*).

However, to take full advantage of the multivariate kernel density (MVKD) approach [35], we computed multivariate LRs that incorporate multiple formants from a single vowel simultaneously. This approach captures the correlations within the raw data, allowing for a more accurate weighting of different parameters to produce a single score for each vowel. These scores were then be fused with scores from other vowels. Both *fusion* and *calibration* are achieved through logistic-regression-based methods, which provide a weighted combination of parallel sets of likelihood ratios derived from different parameters [34, 36]. The calculations of multivariate kernel density LR-like scores, fusion and calibration were done using the R package *fvclrr* [37].

To compute LR-like scores within the same speaking style (i.e., *Dialogue* and *Interview*), speakers’ data was split into reference and questioned portions within each style. For the calculation of LR’s in mismatched speaking styles (i.e., *interview* Vs. *dialogue*), the *interview* data was treated as the reference material and the *dialogue* data was treated as the questioned material.

#### Cross-validation

To compose the background sample, a cross-validation procedure was employed to generate LR-like scores using Multivariate Kernel Density (MVKD) estimation and to calibrate the system. Numerous pairwise comparisons were conducted among non-twin individuals in each speaker group, utilizing a background sample comprising data from all speakers except those directly involved in the comparison. Each time a speaker pair was tested out of the 10 speakers in each group, the remaining speakers in the database served as the reference population. Given that there are 45 possible combinations among 10 speakers and that 150 random speaker groups were chosen, this results in a total of 6,750 pairwise comparisons.

The division of subjects into 150 random speaker groups comprising 10 speakers has both advantages and limitations. One main advantage is ensuring that twin pairs are excluded from both the testing and background samples, resulting in less biased LR-like scores. Another advantage is the ability to compare general outcomes across speaker groups and check for consistency. There are also drawbacks. The main one regards the reduction of the reference population size, which can affect the precision of likelihood ratios (LR) [38].

It should be noted that our present study benefits from data acquired under optimal conditions with high-quality recordings, which inherently leads to *C*_*llr*_ and EER values that may not reflect typical real-life circumstances. However, given the experimental nature of our study, our primary focus is on understanding general speaker discriminatory patterns of vowel formants and the style influence without accounting for the impact of audio quality.

Finally, it is worth highlighting that there will likely be some level of variation in *C*_*llr*_ and EER outcomes across different studies, which has to do with different factors, such as the definition of the relevant population, number of speakers in the reference population, and the amount of data used during tests [38].

Overall, considering the number of possible comparisons across speakers, the number of random speaker groups, the multiple data point random downsampling, the number of formants tested, the analysis performed within each speaking style involved over 5 milion iterations. For mismatched speaking styles, over 10 milion iterations were involved.

#### Assessing speaker discriminatory performance

As previously mentioned, two widely recognized features are deemed crucial for candidate parameters in speaker comparison: low intra-speaker variability and high inter-speaker variability [25]. Ideally, when a pairwise sample is tested against a background sample to assess the typicality of an outcome, the chosen parameters should exhibit minimal intra-speaker variability and substantial inter-speaker variation. Subsequently, the performance of these parameters can be evaluated using discriminatory performance metrics.

For such an assessment, two discriminatory performance metrics were examined to evaluate the discriminatory power of vowel formants in the present study. The first metric is the *Log-likelihood-ratio-cost function* (*C*_*llr*_), an empirical estimate of the precision of likelihood ratios proposed by [39] and applied in several studies [2, 28, 38, 40].

According to [36], the *C*_*llr*_ metric has the desired properties of being based on likelihood ratios, being continuous, and more heavily penalizing worse results (i.e., providing less support for the consistent-with-fact hypothesis or more support for the contrary-to-fact hypothesis). *C*_*llr*_ is calculated through the Formula 2:

$$\begin{array}{c} C_{llr}=\frac{1}{2}(\frac{1}{N_{ss}}\;\sum_{i=1}^{N_{ss}}\;{\text{log}}_{2}[1+\frac{1}{LR_{{ss}_{i}}}]+\frac{1}{N_{ds}}\sum_{j=1}^{N_{ds}}\;{\text{log}}_{2}[1+LR_{{ds}_{j}}]) \end{array}$$ (2)

In Formula 2, *Nss* and *Nds* are the number of same-speaker and different-speaker comparisons, respectively, whereas *LRss* and *LRds* are the likelihood ratios derived from same speaker and different speaker comparisons, respectively. A same-origin penalty value is *log*_2_(1 + 1/*LRs*), and a different-origin penalty value is *log*_2_(1 + *LRd*).

The second estimate is the *Equal Error Rate* (EER), which represents the point where the false reject rate (type I error) and false accept rate (type II error) are equal, being used to describe the overall accuracy of a biometric system [41]. Such a metric is also generated along with the *C*_*llr*_ metric using the R package “*fvclrr*” [37]. EER is calculated through the Formula 3:

$$\begin{array}{c} EER=\frac{1}{2}(\frac{FAR+FRR}{2}) \end{array}$$ (3)

In Formula 3, The FAR is the rate at which the system incorrectly accepts an impostor as the target speaker, while the FRR is the rate at which the system rejects the target speaker incorrectly. By computing the EER, we can determine the point at which the system is equally likely to make both types of errors.

An ideal metric for the forensic application should present relatively low *C*_*llr*_ and EER in relation to the other parameters under comparison. As a rule of thumb, *C*_*llr*_ values closer to 0 suggest better discriminatory performance, whereas a *C*_*llr*_ of 1 indicates a system that outputs likelihood ratios (LRs) of 1 for any comparison, meaning it captures no useful speaker discriminatory information. For EER, values closer to 0.00 indicate better performance, signifying a lower rate of incorrect classifications. Conversely, values approaching 0.50 reflect worse performance, corresponding to a chance level of discrimination where the system is no better than random guessing.

*C*_*llr*_ and EER values were computed for comparisons within random speaker groups and then averaged across all iterations. Given the extensive number of iterations and calculations of discriminatory power metrics reported earlier, *C*_*llr*_ and EER outlier values were automatically identified and removed using the 2.5*IQR rule.

## Results

The focus of this study was to evaluate the discriminatory power of vowel formant frequencies, widely examined parameters in speaker comparisons, across two distinct speaking styles commonly present in such a task: *dialogue* and *interview*. In this section, we present and contextualize our findings.

**Fig 1:**
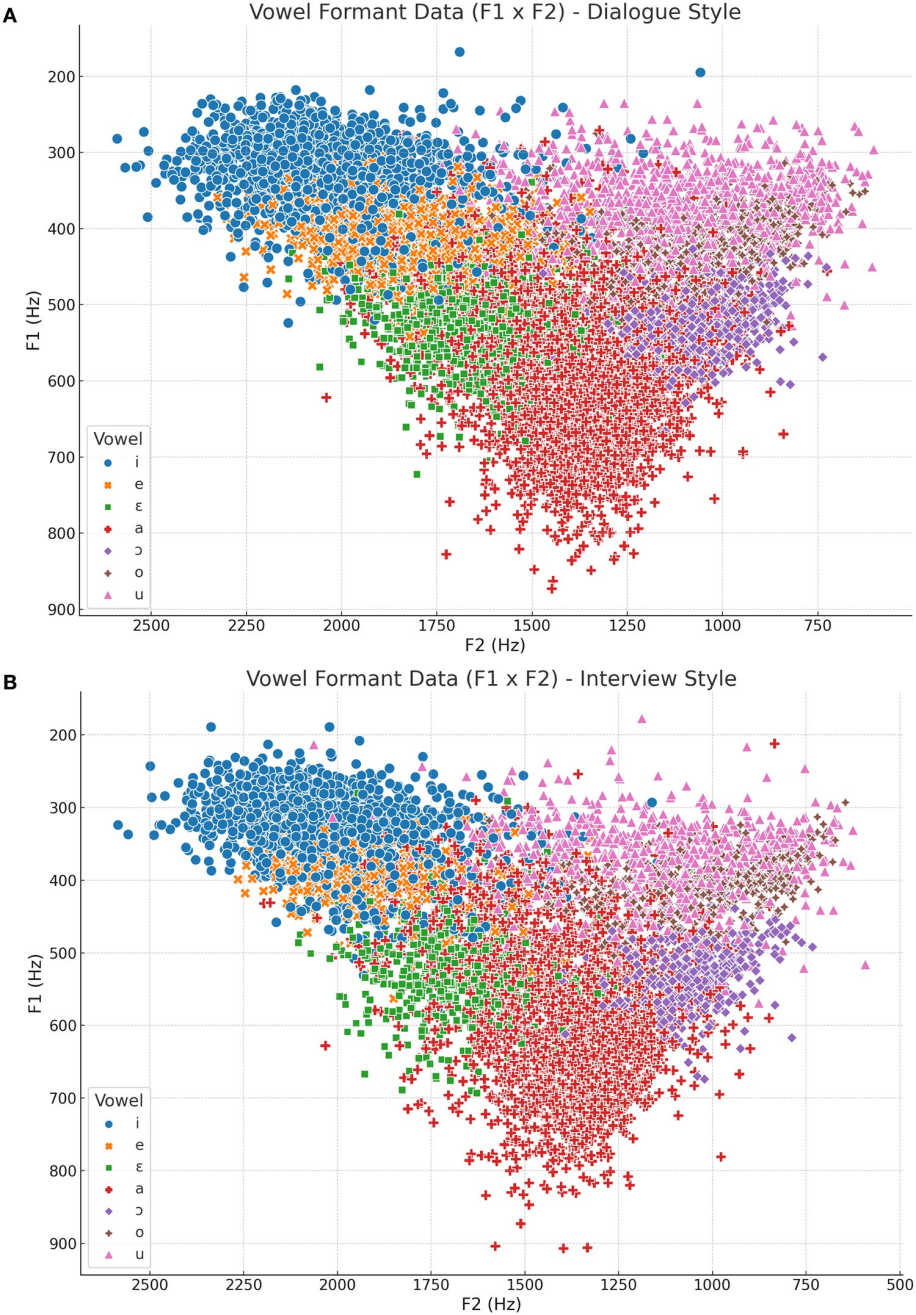
Vowel data points as per vowel quality for dialogue (A) and interview (B) speaking styles based on the F1 x F2 relation.

**Fig 2:**
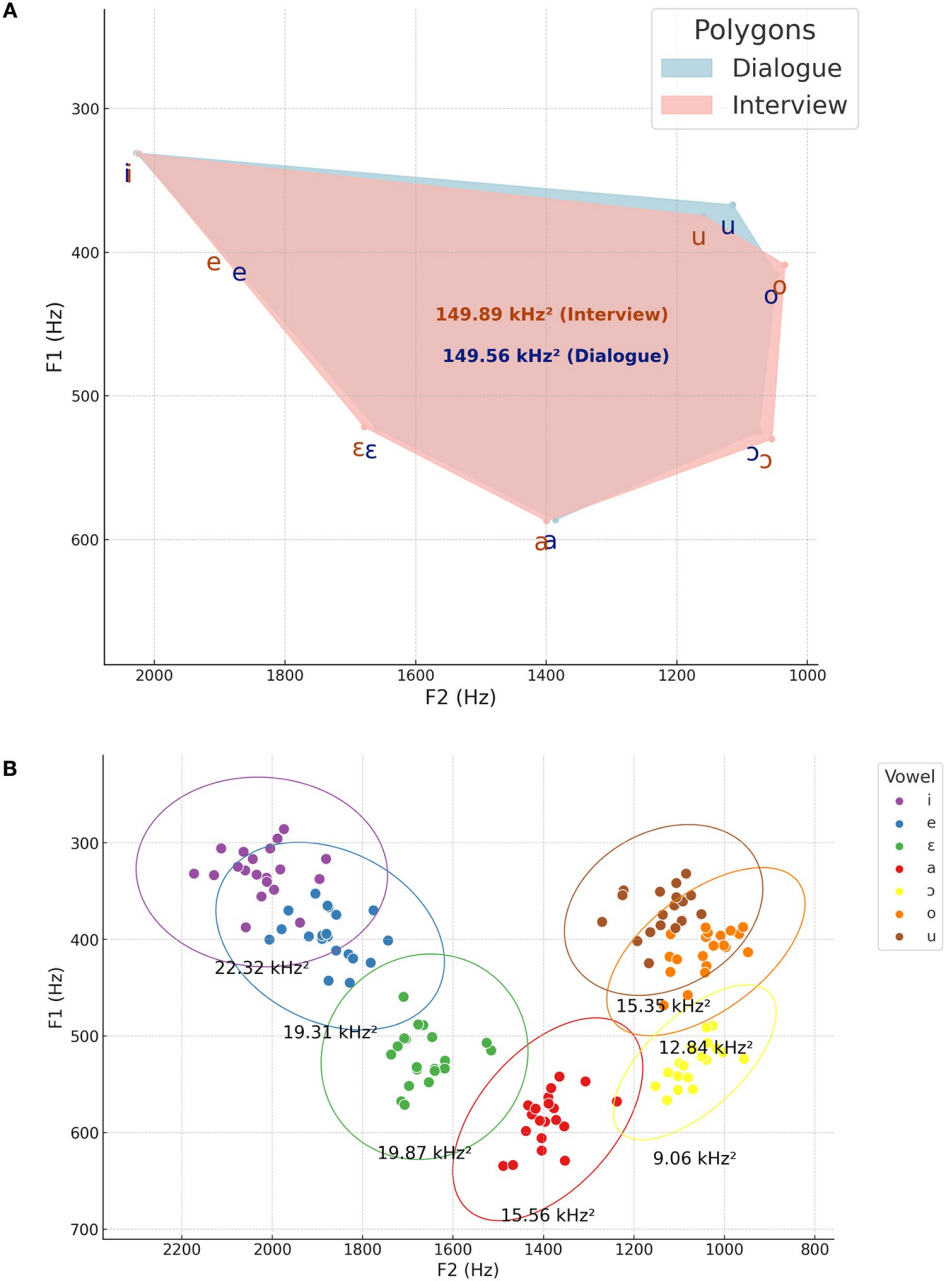
Vowel space polygons for dialogue and interview speaking styles (A) and speakers’ mean formant frequency values for F1 x F2 as per vowel quality (B).

Fig 1 illustrates the distribution of vowel data points in the F1 x F2 two-dimensional plane for dialogue (Fig 1A) and interview (Fig 1B). This visualization highlights the variability in the phonetic realization of different vowels. It serves as the foundation for calculating average values across all speakers for different speaking styles, as shown in Fig 2A, and within individual speakers, as depicted in Fig 2B.

In Fig 2A, vowel space polygons are presented as a function of speaking styles, allowing the overall comparison of their configuration in the two-dimensional vocalic space. Vowel letters stand for average formant values. Surface areas of the vocalic spaces are depicted. Moreover, in Fig 2B, speakers’ average values for F1 x F2 are displayed with ellipses accounting for the 95% confidence interval of each vowel.

**Fig 3:**
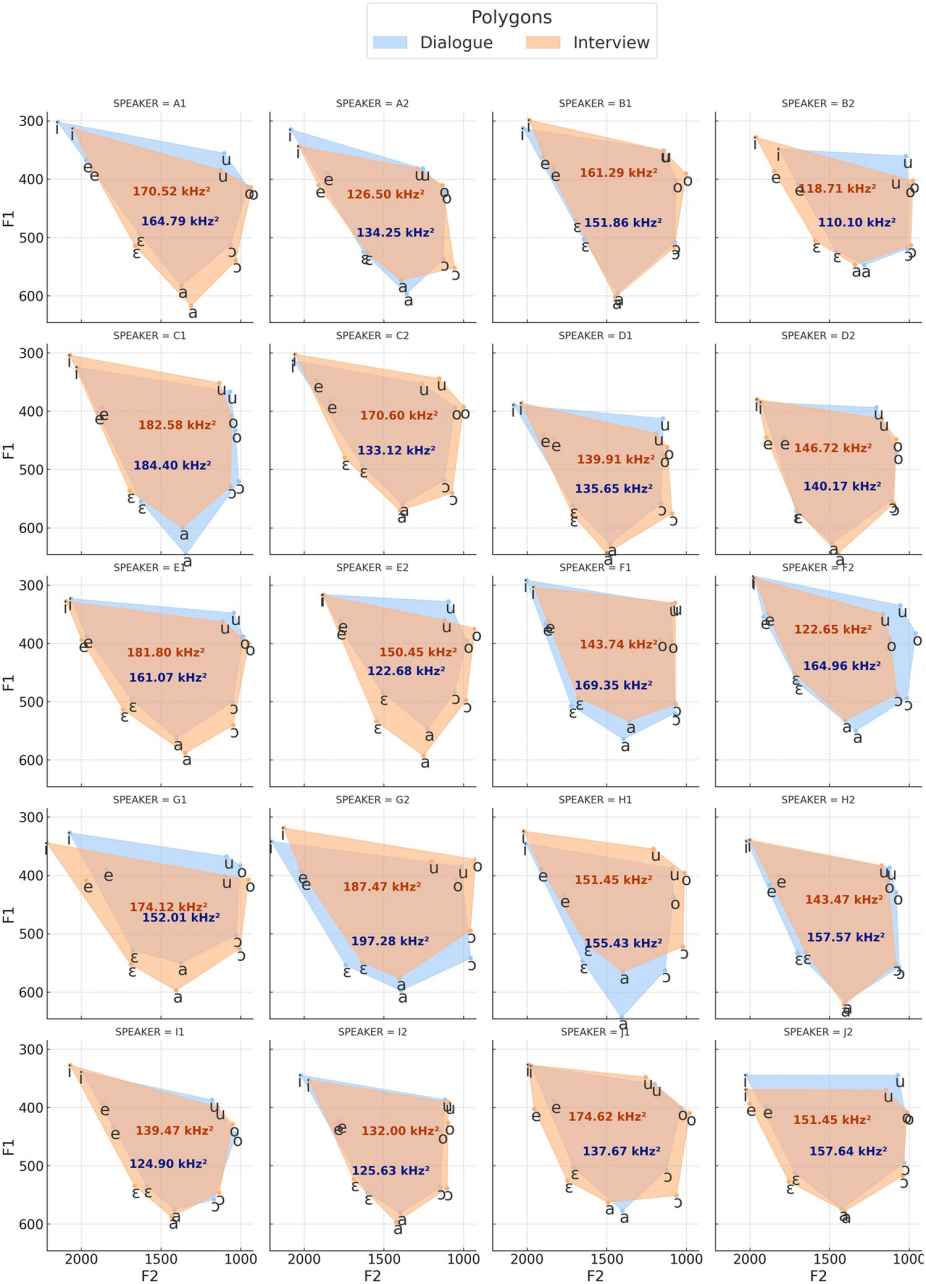
Vowel space polygons for dialogue and interview speaking styles based on F1 x F2 as a function of individual speakers.

**Fig 4:**
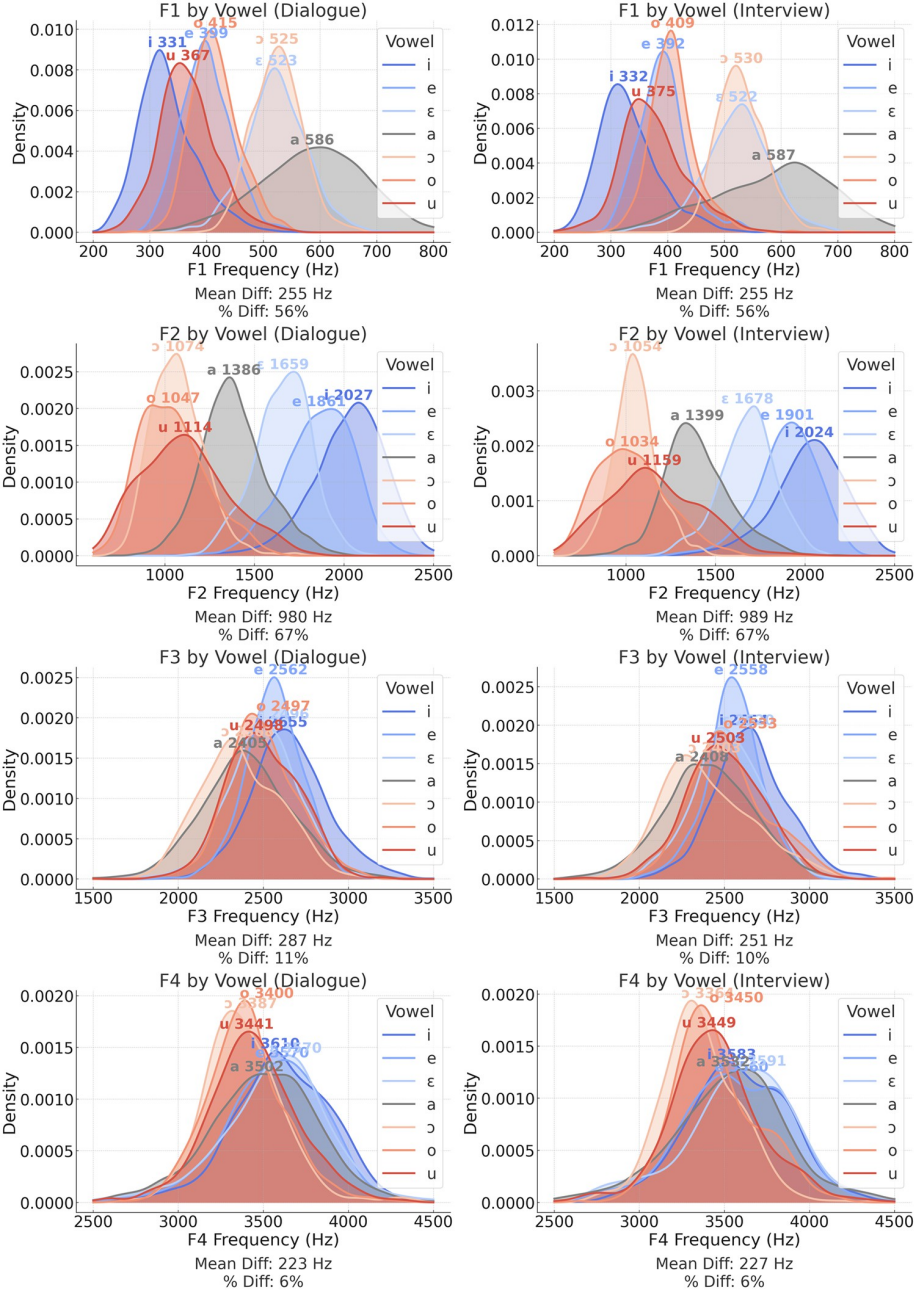
Vowel density distributions for F1, F2, F3, and F4 as per speaking style and vowel quality.

In Fig 3, vowel space polygons are displayed as a function of speaking styles and individual speakers. Surface areas of vocalic spaces are also depicted intra-speakers and intra-speaking styles. In Fig 4, density distribution curves are shown as per vowel quality (vowel type) based on average values of individual formants, i.e., F1, F2, F3, and F4. Those curves show how formant values can vary as per vowel quality. Mean and mean percentual differences as displayed at the bottom of each density distribution plot.

**Fig 5:**
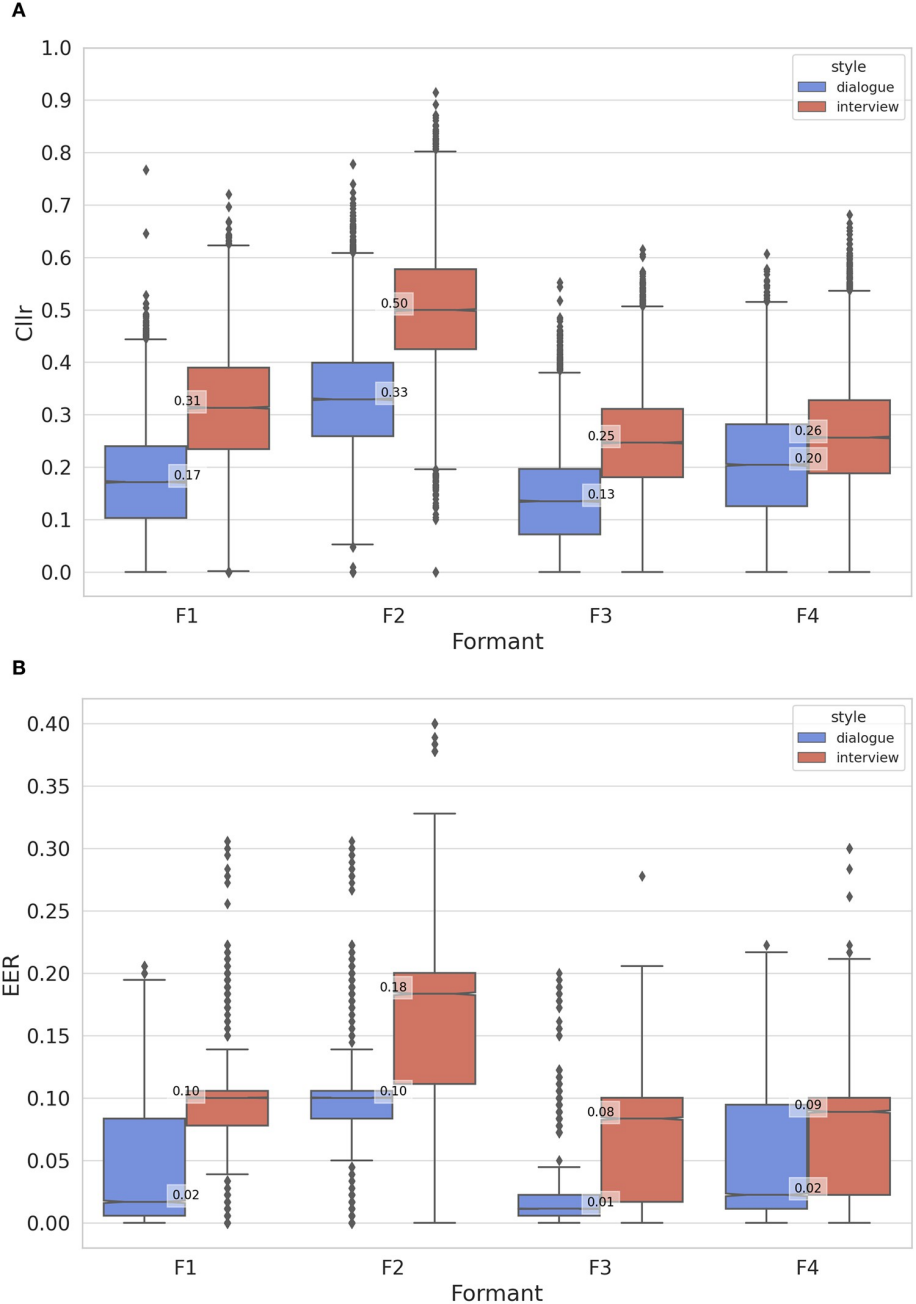
*C*_*llr*_ and EER boxplots for formant frequencies as a function of speaking styles (dialogue and interview).

**Fig 6:**
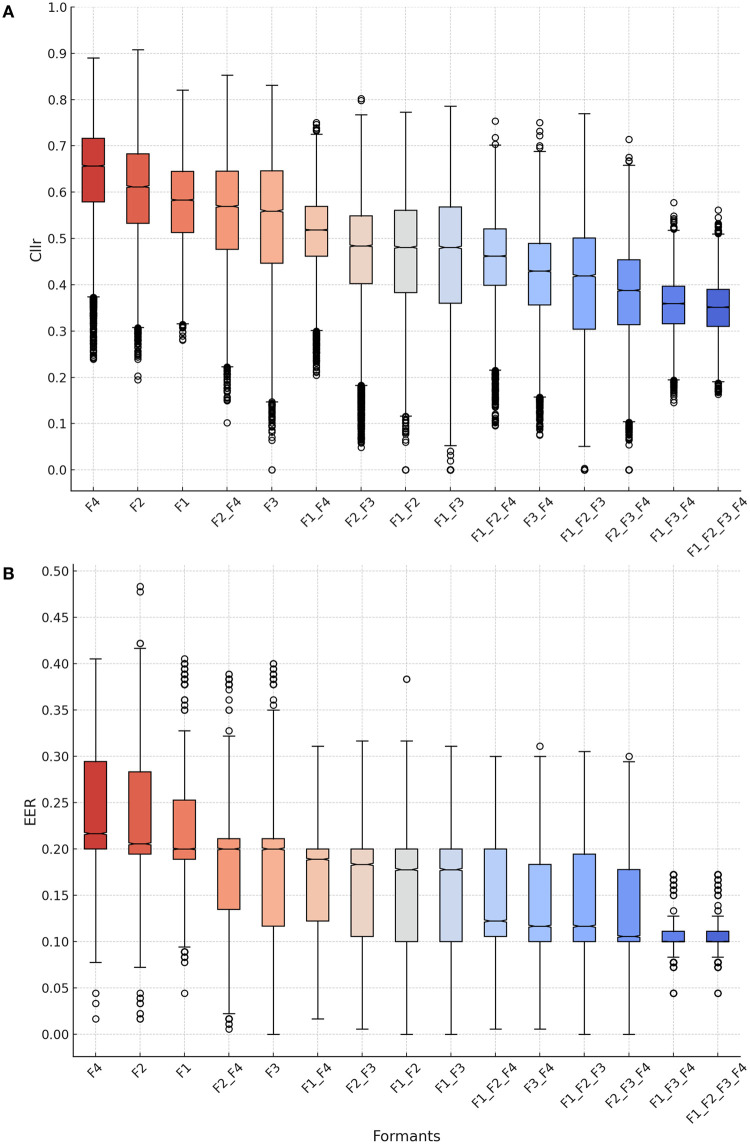
Boxplots for *C*_*llr*_ (A) and EER (B) as a function of formants and formant combinations in mismatched speaking style comparisons: Dialogue Vs. Interview.

In Fig 5, boxplots depict system performance metrics (*C*_*llr*_ and EER) for F1-F4 regarding *dialogue* and *interview* speaking styles. Boxplots in Fig 6 regard the results obtained for mismatched speaking style comparisons involving individual and combined formant frequencies. The observed variation in *C*_*llr*_ and EER along the y-axis in these visualizations is a consequence of the influence of data sampling on the computation of the discriminatory power metrics.

**Fig 7:**
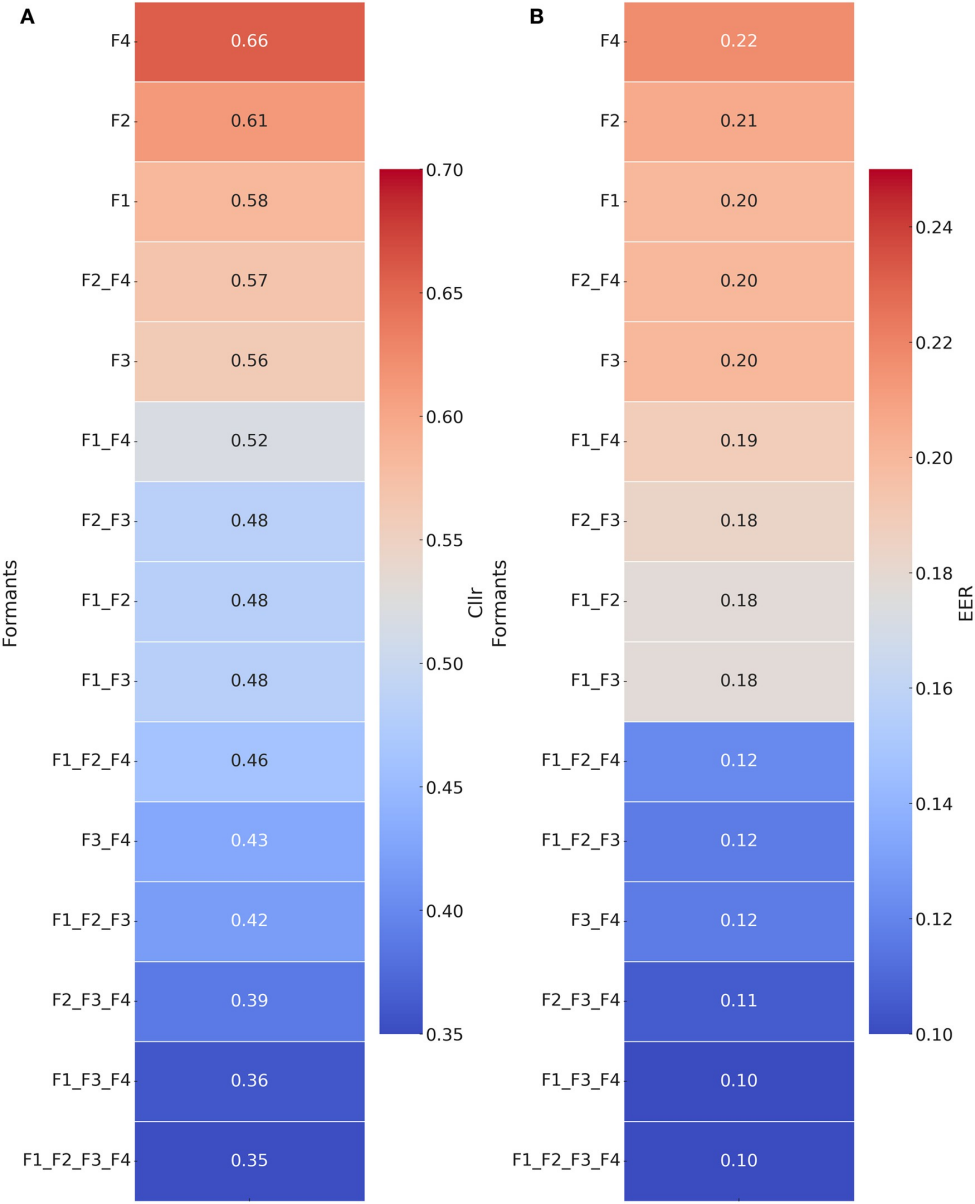
Heatmaps for *C*_*llr*_ and EER as a function of formants and formant combinations in mismatched speaking style comparisons: Dialogue Vs. Interview.

Fig 7 presents heatmaps that help us numerically compare the outcomes presented in Fig 6. The gradient color coding in such heatmaps translates as follows: lower *C*_*llr*_ and EER values are represented by dark blues whereas higher values are represented by dark reds. Differences in average *C*_*llr*_ and EER across parameters are reflected in the values being presented and in the color gradient.

**Fig 8:**
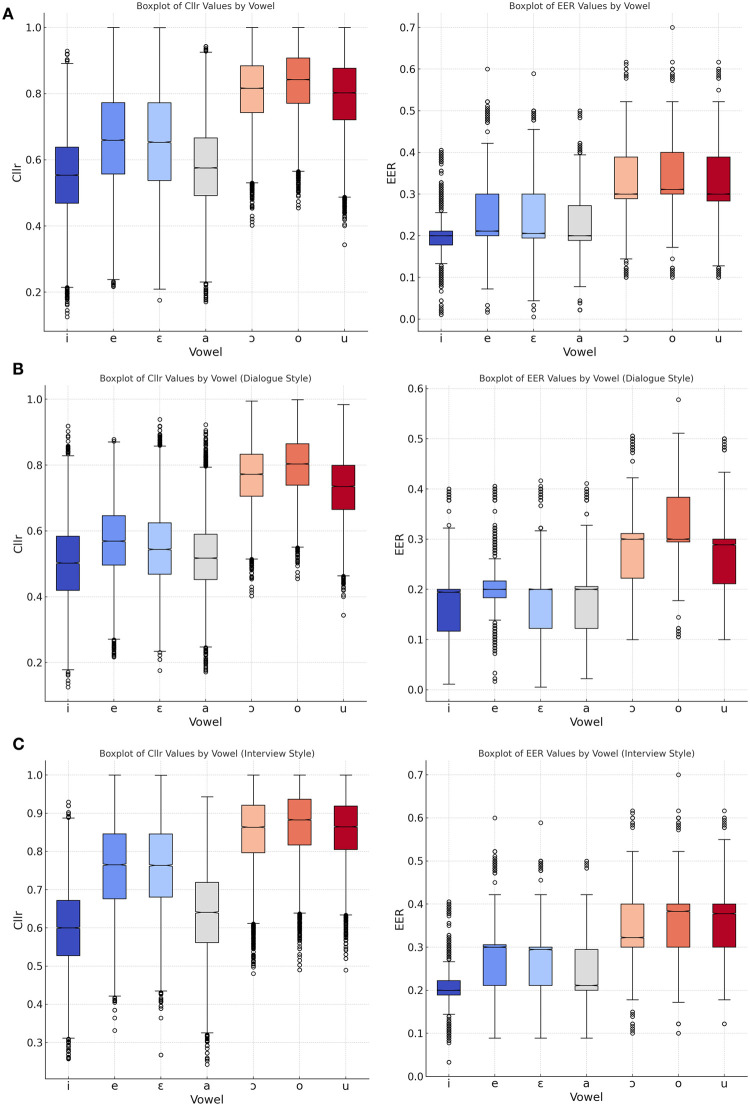
*C*_*llr*_ and EER as a function of vowel quality and speaking styles, combined styles (A), dialogue (B) and interview (C) based on the F1 + F2 combination.

Finally, in Fig 8, a vowel quality-dependent assessment of *C*_*llr*_ and EER values is displayed. The results are focused only on the relation F1 x F2 for intra-speaking style comparisons, as the intention was to compare the discriminatory potential of vowels alone. Fig 8A shows the overall results for vowels without considering specific styles, Fig 8B displays the results for *dialogue*, and Fig 8C for *interview*.

First, we begin with some brief qualitative considerations to help us better understand the overall outcomes. These considerations address the impact of vowel quality on individual formants and the influence of speaking style on the configuration of vocalic spaces. After these qualitative insights, we present the statistical results.

### Dialogue versus interview

As shown in Fig 2A, there is almost a complete overlap between the acoustic spaces of different speaking styles, despite some slight differences in average ‘F1 x F2’ values based on vowel quality. The surface areas of the vowel space polygons for *dialogue* and *interview* are very similar. For some back vowels, particularly the low and high-mid back vowels, the acoustic space is slightly wider in the *interview* style compared to *dialogue*. Conversely, the vowel /u/ appears slightly more centralized in the *interview* style than in *dialogue*.

Despite the overall similarity in vowel space configurations shown in Fig 2A, speaker-specific particularities can still be observed. As depicted in Fig 3, speakers exhibit different patterns in how speaking style affects their vocalic spaces. Visualizing Fig 3 and numerically comparing surface vowel space areas reveals two categories: individuals with a slightly wider acoustic space in the *interview* style compared to *dialogue* (e.g., A1, B1, B2, C2, D1, D2, E1, E2, G1, I1, I2, J1), and those with a slightly wider vowel space area for *dialogue* (e.g., A2, C1, F1, F2, G2, H1, H2, J2). In some cases, there is primarily a displacement of the entire acoustic space either vertically or horizontally.

The observation that different speakers may exhibit distinct visual trends in their vowel space configurations across various speaking styles (Fig 3) might shed some light on the less pronounced differences observed when comparing the acoustic spaces of the general speaker group (Fig 2A), as individual differences may counterbalance each other. However, these trends should be further explored in future analyses dealing primarily with the study of style to probe their statistical and potential linguistic significance.

### Vowel quality and formants

Fig 4 illustrates the impact of vowel quality on the density distribution of F1-F4 formants. It is clear that vowel quality affects the average values of the lower formants in both speaking styles to a greater extent. For example, in the *dialogue* speaking style, the average F1 difference among vowels ranges from approximately 331 Hz to 586 Hz (a difference of 56%), and the average F2 difference ranges from 1.074 Hz to 2.027 Hz (a difference of 67%). The variability of vowels’ F1 x F2 is also evident in Fig 2, allowing us to distinguish between different vowel categories.

Conversely, while differences across formants are generally expected to become more pronounced in higher spectral regions, lower variability was observed for the higher formants (F3 and F4). In the *dialogue* speaking style, F3 values ranged from 2.368 Hz to 2.655 Hz, and F4 values ranged from 3.387 Hz to 3.570 Hz, with differences of approximately 10% and 6%, respectively. If we had a visual representation of the F3 x F4 relationship (as in Fig 2B), it would show vowel points centered around specific locations in the two-dimensional acoustic space.

Having made some qualitative remarks on the data, we now address the statistical outcomes concerning the analysis of the speaker discriminatory power of formants as a function of speaking style and vowel quality.

### The discriminatory power of vowel formants

When inspecting the *C*_*llr*_ and EER boxplots in Fig 5 for individual formant frequencies assessed in style-matched conditions, it was evident that F3, F4, and F1 (in order of relevance) were the best-performing formants in both speaking styles in most cases. Notably, F2, when assessed in isolation, demonstrated lower speaker discriminatory performance. When narrowing down the analysis to compare results between styles, it is possible to observe that system performance was higher for dialogue in relation to interview as demonstrated by lower *C*_*llr*_ and EER values in Fig 5A and in Fig 5B, respectively.

Regarding mismatched speaking style analyses, Fig 6 illustrates *C*_*llr*_ and EER scores for individual and combined parameters. Comparing F1-F4 boxplots in this figure with F1-F4 boxplots in Fig 5 reveals that discriminatory performance is higher in style-matched conditions. Notably, average *C*_*llr*_ and EER scores shifted towards higher values in Fig 6. It is evident that all formants are affected by style, with F3 showing a slightly lower median *C*_*llr*_ (Fig 6A) and a larger distribution towards lower EER values (Fig 6B).

However, it is worth noting that the combined discriminatory power of F3 and F4 is higher than that of F1 and F2, as indicated by lower *C*_*llr*_ and EER median values in Fig 6. It is safe to say that multivariate analysis is the most recommended approach when dealing with mismatched speech materials. Combining different formants proved to be the best method for modeling speaker differences. Even the combination of F1 and F2, which performed the least effectively among all formant combinations, outperformed the best-performing single variable, F3, in Fig 6.

As can be observed in Figs 6 and 7, the combination of higher formants ‘F3 + F4’ seemed to outperform the combination of lower formants ‘F1 + F2’, as suggested by lower *C*_*llr*_ and EER median values. For instance, *C*_*llr*_ scores decreased from 0.49 to 0.39, and EER scores decreased from 0.19 to 0.11 when comparing ‘F3 + F4’ against ‘F1 + F2’.

In the multivariate analysis, it was possible to verify that the magnitude of improvement in *C*_*llr*_ and EER scores increased as more formants were incorporated into the model, especially when high formants (i.e., F3 and F4) were included. Notably, the best discriminatory performance was achieved when most formants were combined. However, removing or adding F2 from the final model did not impact system performance substantially. As can be observed by comparing results between models (‘F1 + F3 + F4’ vs. ‘F1 + F2 + F3 + F4’) in Fig 7, the differences were minimal.

As shown in Fig 7, when all formants were included in the model, *C*_*llr*_ and EER scores dropped to 0.35 and 0.10, respectively, in relation to the best performing single formant, i.e., F3 (*C*_*llr*_ = 0.56, EER = 0.20). The overall *C*_*llr*_ and EER distributions were also shifted, revealing a score variability within lower *C*_*llr*_ and EER ranges in Fig 6.

Concerning the assessment of discriminatory power as a function of vowel quality based on the formant relation ‘F1 x F2’, a general pattern was suggested in Fig 8. Overall, front vowels tended to outperform back vowels in terms of their discriminatory power. Front vowels and central vowel /a/ tended to depict the lowest *C*_*llr*_ and EER scores when looking at the overall results without considering specific styles in Fig 8A, and when narrowing down the analysis to *dialogue* in Fig 8B, and *interview* in Fig 8C.

The dispersion and variability of different vowels were presented in Fig 2B. By inspecting this figure, one can observe that front vowels have larger ellipse areas compared to back vowels. As already mentioned, these areas represent the 95% confidence interval for speakers’ average F1 x F2 values when data from both *dialogue* and *interview* speaking styles are combined. The possible relationship between vowel dispersion and discriminatory power will be addressed in the discussion.

For reasons already explored, namely, the higher impact of articulatory constraints on lower formants [6, 7] (see Fig 4), only F1 and F2 were considered for the assessment of the discriminatory power of specific vowels in Fig 8. The outcomes may not be generalized to conditions where higher formants, such as F3 and F4, are incorporated into the models. However, for making a linguistic sense of the differences in performance among vowels, an ‘F1 x F2’-based comparison suffices. Regarding the potential forensic implications of the present analysis, it is preferred to refer to Fig 6, where all vowels’ combined discriminatory power is considered as per different formant combinations.

## Discussion

In this follow-up study to [3], we aimed to assess the speaker discriminatory power of vowel formants in two different speaking styles: *Dialogue* and *Interview*. We applied a likelihood ratio-based statistical approach and included both style-matched and mismatched speaking styles in the analysis. This differs from the previous experiment by also examining the implications of using different speech data as ‘reference’ and ‘questioned’ materials on the outcomes.

Some previous hypotheses and findings from [3] have been corroborated and further explored in this paper. Regarding the comparison across all subjects in the corpus, F3 and F4, along with F1, were found to contain a higher level of speaker-specific information that could be effectively exploited by the system. When comparisons in mismatched speaking styles were made, higher formants (F3 and F4) tended to present better overall discriminatory performance than lower formants (F1 and F2), but only when assessed in combination. These findings align with previous reports in the literature [4, 42, 43], suggesting that higher formants may carry more speaker-specific information than lower formants when controlling for style.

As discussed in [3], the control of the linguistic component on the limits of variation for vowel production, justified for articulatory reasons, suggests that less speaker-discriminatory information is expected in formants that are under stronger linguistic constraints. This impact appears to be particularly significant for F2, which is closely related to vowel place of articulation and serves as an acoustic indicator of the constriction position in vowel production [7]. Consequently, differences in F1 across speakers seem to be more tolerated than differences in F2 values for the same vowels. It remains to be explored whether languages that rely heavily on differences in F3 to contrast front-rounded and unrounded vowels— such as Swedish— will present similar patterns.

For higher formants, such as F3 and F4, lower linguistic constraints may be presumed. This observation leads to the hypothesis that variations in the resonance of higher formants, associated with vocal tract configurations and individual phonatory settings, may be more speaker-discriminatory within styles. However, if speakers are expected to adjust both the phonetic realization of their vowels and their vocal tract configurations based on speaking style, then it is expected that speaking style will impact all formants to some degree.

Another observation regards the relatively higher speaker discriminatory power of front and central vowels compared to back vowels as far as the ‘F1 x F2’ relation is concerned. This finding aligns with our previous experiment [3], and experiments conducted in other languages, such as Australian English [44]. A combination of factors may account for such an observation. In [3], it was observed that front and central vowels displayed the largest Euclidean distances among them, which may partially explain the higher variability observed for these vowels. The possibly lower levels of variation allowed for back vowels, compared to front vowels, could be due to discrepancies in articulatory working space. Increased proximity between vowels due to alternative articulatory realizations could imply perceptual difficulties. Another important factor to be considered regards the concept of “sufficient contrast” [45], meaning the phonetic distance between different vowels, may explain why some vowels, although not displaying the highest acoustic distances from their neighbors may still allow more variation within front and back dimensions (e.g., /i/ and /u/). As for the /a/ vowel, the combination of these two factors may position it favorably for higher speaker discrimination. In terms of vocalic dispersion, /a/ is the only central vowel in the BP system and also the one that tends to display the greatest distances from its neighbors [3].

It is also likely that the disparity in the number of vowel tokens contributed to the observed asymmetry in discriminatory power. As previously noted, the most frequent vowels in descending order were: /a/, /i/, /u/, / ε/, /e/, /o/, and / ͻ/. However, the fact that /u/ was the third most frequent but less discriminatory than / ε/ and /e/ indicates that other factors are also influencing this outcome. Furthermore, as highlighted in [3], the higher frequency of occurrence of certain tokens can itself justify the selection of these vowels for speaker comparison purposes. Thus, *availability* is a critical factor, especially in situations where substantial data is lacking for analysis.

Regarding the discriminatory power of formants in mismatched speaking style comparisons, the findings emphasize the importance of a multivariate analysis that integrates multiple formants, thereby capturing speaker-specific information embedded within them. Generally, stronger discriminatory performance was achieved when multiple formants for all vowels were considered, reducing the uncertainty in likelihood ratio calculations. This suggests that, although higher formant frequency regions may contain more speaker-specific information, the cumulative contribution of speaker-specific information across different formants can enhance discriminatory performance at various levels. This seems particularly crucial when intra-speaker variability increases by the influence of different factors, such as style.

Evaluating higher formant frequencies has long been considered unfeasible due to the limitations imposed by telephone bandwidth [46, 47]. This could explain the lack of research on these formants. However, as pointed out by [4] and reinforced in [3], voice communication via means other than telephone calls has become increasingly common, such as through cross-platform messaging apps (e.g., WhatsApp, Telegram), which allow users to exchange voice messages. For instance, in Brazil [48], direct telephone calls have become increasingly uncommon and communication through voice messages has become the new standard, which has created an opportunity to investigate a novel set of acoustic descriptors that were previously unexplored. Therefore, scientific knowledge must progress in tandem with technological advances, and thorough testing of these descriptors is necessary to determine their viability.

Regarding the specific impact of speaking style on vowel acoustic space, a qualitative analysis of the data suggests different patterns that may be better explained by speaker-dependent trends. While some individuals exhibited a higher dispersion of vowels based on F1 or/and F2—and therefore a larger acoustic space surface area—in interviews, others displayed the opposite trend. This apparent speaker-dependent factor may help explain the divergences observed in previous literature. A more homogeneous pattern is observed when comparisons of acoustic vowel spaces in different speaking styles are made without considering the ‘speaker’ factor, as shown in Fig 2A.

These observations are important, given the lack of more controlled research on the impact of the comparison between *Dialogue* vs. *Interview* speaking styles and changes on vowel acoustic space configurations. Most studies focusing on telephone/mobile phone-transmitted speech have been dedicated to assessing the artificial impact of the transmission system on specific formants and vowels, e.g., [23, 46].

A widespread assumption in studies on vowel variability regards the idea that the vowel acoustic space tends to be less dispersed in spontaneous speech than in elicited speech [15, 49, 50], which is also accompanied by a greater intra-vowel variability. These phonetic differences across styles do not seem to be entirely related to durational differences, as speakers also appear to adjust their articulatory precision following style [49].

The study by [50] demonstrated that words with many phonological neighbors tend to be phonetically reduced in connected speech when other factors influencing word duration are controlled for. Such words are often shortened and produced with articulatory undershoot, resulting in more centralized vowels. According to the authors, their findings are fully compatible with the idea that variation at certain levels of linguistic structure, with different degrees of planning and encoding, may reflect speakers’ models of their listeners and their surrounds. Speakers take into account the needs of their listeners, and this is reflected in their production.

Despite the general assumption of a less dispersed vowel space in spontaneous speech compared to elicited speech [15, 49, 50], this trend does not seem to apply to all speakers in the present study. It remains unclear why different speakers exhibit varying or even opposite patterns in the acoustic configuration of their vowels across speaking conditions. Although identifying the underlying factors contributing to the variation in vowel acoustic spaces was not the primary aim of our study, but rather how this variation affects speaker discriminatory performance, we present a hypothesis that merits exploration in future experiments.

One question worth considering is whether certain speakers modify their vowel articulation and acoustic patterns to compensate for the absence of visual cues and limited bandwidth in mobile phone-transmitted speech. Such adjustments may enhance the auditory acoustic differences between vowels during spontaneous dialogues, leading to varying trends in vowel space configuration across styles. In other words, speakers may employ unique strategies for maintaining intelligibility and expressiveness across different speaking conditions, leading to diverse patterns of vowel space configuration. These individual strategies could be influenced by factors such as personal communication style and familiarity with the task. This aligns with the notion that variation may reflect speakers’ models of their listeners and their surrounds [50]. If this hypothesis holds, it may explain the speaker-dependent trends observed here, as some may be inadvertently attempt to produce a register closer to what can be termed “clear speech” [18].

While further research is necessary to test the aforementioned hypothesis, studies on other acoustic-phonetic descriptors, such as the voice fundamental frequency (*f*0), have shown that speakers may be aware of the constraints of telephone transmission and adjust their average estimates accordingly, depending on whether they are communicating face-to-face or over the phone [51]. It is essential to consider the potential implications of these adjustments for speaker discrimination. If some speakers are more prone to adapting to the constraints of telephone transmission, this could result in different discrimination rates across speakers and potentially lead to errors or biases in speaker comparisons.

## Conclusion

In style-matched analysis of the discriminatory power of vowel formants, it has been verified that F3, F4, and F1 (in order of importance) were the best-performing formants in both speaking styles assessed. F2 was the parameter that presented a weaker speaker discriminatory performance in most cases. In general, better discriminatory performance was obtained for *Dialogue* in relation to *Interview*. This difference could be attributed to higher speaker variability in spontaneous dialogues between very familiar speakers. The fact that more data was available for *Dialogue* compared to the *Interview* could also have influenced the results, possibly potentiating the differences.

It was confirmed that comparing speakers in mismatched speaking styles (*Interview* vs. *Dialogue*) had an impact on speaker discriminatory performance. When comparing individual formants (F1-F4), the average *C*_*llr*_ and EER scores shifted towards higher values. It is evident that all formants are affected by speaking style, with F3 displaying a slightly lower median *C*_*llr*_ and a wider distribution towards lower EER values. Under these conditions, the combination of higher formants (F3 and F4) demonstrated higher discriminatory performance than the combination of lower formants (F1 and F2), which indicates a better separation across subjects.

Regarding mismatched speaking styles, as expected, it was observed that the magnitude of improvement in *C*_*llr*_ and EER scores increased as more formants were incorporated into the models, particularly with the addition of higher formant frequencies (i.e., F3 and F4). The best discriminatory performance was achieved when most formants were combined.

Regarding the assessment of discriminatory power as a function of vowel quality and the ‘F1 + F2’ formant relation, a pattern previously observed in earlier analyses was suggested. Front vowels and the central vowel tended to exhibit the lowest *C*_*llr*_ and EER scores in the tested speaking styles. As discussed in a previous study [3], various factors may influence the level of speaker-specific information present in different vowels, including vowel dispersion, variability and distinctiveness. The disparity in the number of vowel tokens may also have contributed to such an outcome.

Finally, it is possible that speakers adjust their vowel articulation to compensate for the absence of visual cues and limited bandwidth in mobile phone-transmitted speech, which may result in varying trends across speaking styles. These adjustments could be influenced by individual communication strategies, familiarity with the task or other underlying factors.

## Supporting information

S1 Data (TXT)

## References

[1] Gold Erica, French Peter. International practices in forensic speaker comparison. International Journal of Speech, Language and the Law, 18(2):293–307, 2011. doi: 10.1558/ijsll.v18i2.293

[2] Cavalcanti Julio Cesar, Eriksson Anders, Barbosa Plínio A. On the speaker discriminatory power asymmetry regarding acoustic-phonetic parameters and the impact of speaking style. Frontiers in Psychology, volume 14, 2023. doi: 10.3389/fpsyg.2023.1101187 37138997 PMC10150585

[3] Cavalcanti Julio Cesar, Eriksson Anders, Barbosa Plínio A. Acoustic analysis of vowel formant frequencies in genetically-related and non-genetically related speakers with implications for forensic speaker comparison. Plos One, volume 16(2), page e0246645, 2021. Public Library of Science San Francisco, CA USA. doi: 10.1371/journal.pone.0246645 33600430 PMC7891727

[4] Cao Honglin, Dellwo Volker. The role of the first five formants in three vowels of mandarin for forensic voice analysis. International Congress of Phonetic Sciences, pages 617–621, 2019.

[5] Ladefoged Peter, Broadbent Donald Eric. Information conveyed by vowels. The Journal of the Acoustical Society of America, 29(1):98–104, 1957. doi: 10.1121/1.1908694 2525139

[6] Rose Philip. Forensic Speaker Identification. Tayler Francis, volume 3, 54–67, 2002.

[7] Stevens Kenneth N., House Arthur S. Development of a quantitative description of vowel articulation. The Journal of the Acoustical Society of America, 27(3):484–493, 1955. doi: 10.1121/1.1907943

[8] Sundberg, Johan. Ciência da voz: fatos sobre a voz na fala e no canto. Editora da Universidade de São Paulo, 2015. ISBN: 9788531415104.

[9] Traunmüller Hartmut. Articulatory and perceptual factors controlling the age- and sex-conditioned variability in formant frequencies of vowels. Speech Communication, 3(1):49–61, 1984. doi: 10.1016/0167-6393(84)90008-6

[10] Takemoto Hironori, Adachi Seiji, Kitamura Tatsuya, Mokhtari Parham, Honda Kiyoshi. Acoustic roles of the laryngeal cavity in vocal tract resonance. The Journal of the Acoustical Society of America, 120(4):2228–2238, 2006. doi: 10.1121/1.2261270 17069318

[11] Takemoto Hironori, Mokhtari Parham. Acoustic analysis of the vocal tract during vowel production by finite-difference time-domain method. The Journal of the Acoustical Society of America, 128(6):3724–3738, 2010. doi: 10.1121/1.3502470 21218904

[12] Escudero Paola, Boersma Paul, Rauber Andréia Schurt, Bion Ricardo A. H. A cross-dialect acoustic description of vowels: Brazilian and European Portuguese. The Journal of the Acoustical Society of America, volume 126(3), pages 1379–1393, 2009. doi: 10.1121/1.3180321 19739752

[13] Kent Raymond D., Vorperian Houri K. Static measurements of vowel formant frequencies and bandwidths: A review. Journal of Communication Disorders, volume 74, pages 74–97, 2018. Elsevier. doi: 10.1016/j.jcomdis.2018.05.004 29891085 PMC6002811

[14] Tsao Ying-Chiao, Weismer Gary, Iqbal Kamran. The effect of intertalker speech rate variation on acoustic vowel space. The Journal of the Acoustical Society of America, 119(2):1074, 2006. doi: 10.1121/1.2149774 16521769

[15] Harmegnies Bernard, Poch-Olivé Dolors. Formant frequencies variability in French vowels under the effect of various speaking styles. Le Journal de Physique IV, 4(C5):C5–509, 1994. 10.1051/jp4:19945108

[16] Dankovičová Jana, Nolan Francis. Some acoustic effects of speaking style on utterances for automatic speaker verification. Journal of the International Phonetic Association, volume 29(2), pages 115–128, 1999. Cambridge University Press. doi: 10.1017/S0025100300006496

[17] Heeringa W.J., Schoormann Heike, Peters Jörg. Acoustic cues to vowel identification: The case of /i i/ and /u u/ in Saterland Frisian. Us Wurk. Tydskrift foar frisistyk, 66(1-2):27–76, 2017.

[18] Hargus Ferguson Sarah, Quené Hugo. Acoustic correlates of vowel intelligibility in clear and conversational speech for young normal-hearing and elderly hearing-impaired listeners. The Journal of the Acoustical Society of America, 135(6):3570–3584, 2014. doi: 10.1121/1.4874596 24907820 PMC4048446

[19] Rogers Catherine L., DeMasi Teresa M., Krause Jean C. Conversational and clear speech intelligibility of /bVd/ syllables produced by native and non-native English speakers. The Journal of the Acoustical Society of America, 128(1):410–423, 2010. doi: 10.1121/1.3436523 20649235 PMC2921438

[20] Kahloon Lilah, Shorey Anya E., King Caleb J., Stilp Christian E. Clear speech promotes speaking rate normalization. The Journal of the Acoustical Society of America, 152(4 Supplement):A176–A176, 2022. doi: 10.1121/10.0015946

[21] Koenig Laura L., Fuchs Susanne. Vowel formants in normal and loud speech. Journal of Speech, Language, and Hearing Research, 62(5):1278–1295, 2019. ASHA. doi: 10.1044/2018_JSLHR-S-18-0043 31084509

[22] Passetti, Renata Regina. Estudo acústico-perceptual do estilo de fala telefônico com implicações para a verificação de locutor em português brasileiro. PhD thesis, Universidade Estadual de Campinas (UNICAMP), Instituto de Estudos da Linguagem, 2018.

[23] Byrne Catherine, Foulkes Paul. The’mobile phone effect’ on vowel formants. International Journal of Speech Language and the Law, volume 11(1), pages 83–102, 2007. https://www.equinoxpub.com/journals/index.php/IJSLL/article/view/540. doi: 10.1558/sll.2004.11.1.83

[24] Passetti, Renata Regina, Barbosa, Plínio Almeida. O efeito do telefone celular no sinal da fala: uma análise fonético-acústica com implicações para a verificação de locutor em português brasileiro. Anais do Congresso Brasileiro de Prosódia, number 3, 2015.

[25] Nolan, Francis. The phonetic bases of speaker recognition. Cambridge University Press, 1983. ISBN: 0521244862.

[26] Cavalcanti Julio Cesar, Eriksson Anders, Barbosa Plínio A. Multi-parametric analysis of speaking fundamental frequency in genetically related speakers using different speech materials: some forensic implications. Journal of Voice, 2021. Elsevier. 10.1016/j.jvoice.2021.08.013 34629229

[27] Cavalcanti Julio Cesar, Eriksson Anders, Barbosa Plínio A. Multi-parametric analysis of speech timing in inter-talker identical twin pairs and cross-pair comparisons: Some forensic implications. Plos One, volume 17(1), page e0262800, 2022. Public Library of Science San Francisco CA USA. doi: 10.1371/journal.pone.0262800 35061853 PMC8782339

[28] Cavalcanti Julio Cesar, da Silva Ronaldo Rodrigues, Eriksson Anders, Barbosa Plinio A. Exploring the performance of automatic speaker recognition using twin speech and deep learning-based artificial neural networks. Frontiers in Artificial Intelligence, 7:1287877, 2024. doi: 10.3389/frai.2024.1287877 38405218 PMC10885345

[29] San Segundo, Eugenia. Forensic speaker comparison of Spanish twins and non-twin siblings: A phonetic-acoustic analysis of formant trajectories in vocalic sequences, glottal source parameters and cepstral characteristics. Universidad internacional Menéndez Pelayo, pages 1–318, 2014.

[30] Boersma, Paul, Weenink, David. Praat: doing phonetics by computer [Computer program]. http://www.praat.org/ 2018

[31] Barbosa, Plínio Almeida. ForensicDataTracking [Praat script]. 2022.

[32] R Core Team. R: A Language and Environment for Statistical Computing. R Foundation for Statistical Computing, Vienna, Austria, 2021. https://www.R-project.org/.

[33] Cavalcanti, Julio Cesar, Eriksson, Anders, Barbosa, Plínio A. Assessing the speaker discriminatory power asymmetry of different acoustic-phonetic parameters. 4th International Symposium on Applied Phonetics, 2022. 10.21437/ISAPh.2022-2.

[34] Morrison, Geoffrey Stewart, Zhang, Cuiling, Enzinger, Ewald. Forensic speech science. Thomson Reuters, 2019.

[35] Aitken Colin G.G., Lucy David. Evaluation of trace evidence in the form of multivariate data. Journal of the Royal Statistical Society: Series C (Applied Statistics), volume 53(1), pages 109–122, 2004. Wiley Online Library.

[36] Morrison Geoffrey Stewart, Zhang Cuiling, Rose Philip. An empirical estimate of the precision of likelihood ratios from a forensic-voice-comparison system. Forensic Science International, volume 208(1-3), pages 59–65, 2011. Elsevier. doi: 10.1016/j.forsciint.2010.11.001 21131149

[37] Lo, Justin. fvclrr: Likelihood Ratio Calculation and Testing in Forensic Voice Comparison [R package], version 1.1.1. https://github.com/justinjhlo/fvclrr, 2020.

[38] Hughes, Vincent. The definition of the relevant population and the collection of data for likelihood ratio-based forensic voice comparison. PhD dissertation, University of York, UK, 2014.

[39] Brümmer Niko, Du Preez Johan. Application-independent evaluation of speaker detection. Computer Speech & Language, volume 20(2-3), pages 230–275, 2006. Elsevier.

[40] Morrison Geoffrey Stewart. Forensic voice comparison and the paradigm shift. Science & Justice, volume 49(4), pages 298–308, 2009. Elsevier. doi: 10.1016/j.scijus.2009.09.002 20120610

[41] Conrad, Eric, Misenar, Seth, Feldman, Joshua. CISSP study guide. Newnes, 2012.

[42] Loakes, Deborah. A forensic phonetic investigation into the speech patterns of identical and non-identical twins. 15th International Congress of Phonetic Sciences (ICPhS-15), volume 15, pages 691–694, 2003. https://www.internationalphoneticassociation.org/icphs-proceedings/ICPhS2003/p15_0691.html.

[43] Gold Erica, French Peter, Harrison Philip. Examining long-term formant distributions as a discriminant in forensic speaker comparisons under a likelihood ratio framework. Proceedings of Meetings on Acoustics ICA2013, volume 19(1), page 060041, 2013. Acoustical Society of America. doi: 10.1121/1.4800285

[44] Loakes, Deborah. Front Vowels as Speaker-Specific: Some Evidence from Australian English. Proceedings of the 10th Australian International Conference on Speech Science & Technology, pages 289–294, 2004. https://assta.org/proceedings/sst/2004/proceedings/papers/sst2004-375.pdf.

[45] Lindblom, Björn. Explaining phonetic variation: A sketch of the H&H theory. In Speech production and speech modelling, pages 403–439. Springer, 1990.

[46] Künzel Hermann J. Beware of the ‘telephone effect’: the influence of telephone transmission on the measurement of formant frequencies. Forensic Linguistics, volume 8(1), pages 80–99, 2001. 10.1558/ijsll.v8i1.80

[47] Nolan Francis. The ‘telephone effect’ on formants: a response. Forensic Linguistics, volume 9(1), pages 74–82, 2002. 10.1558/ijsll.v9i1.74

[48] Passetti, Renata Regina, Madureira, Sandra, Barbosa, Plínio Almeida. Voice perception on a voice messaging app: implications for Forensic Phonetics. Proc. Speech Prosody 2022, pages 490–494, 2022. 10.21437/SpeechProsody.2022-100.

[49] DiCanio Christian, Nam Hosung, Amith Jonathan D., García Rey Castillo, Whalen Douglas H. Vowel variability in elicited versus spontaneous speech: Evidence from Mixtec. Journal of Phonetics, volume 48, pages 45–59, 2015. Elsevier. 10.1016/j.wocn.2014.10.003

[50] Gahl Susanne, Yao Yao, Johnson Keith. Why reduce? Phonological neighborhood density and phonetic reduction in spontaneous speech. Journal of Memory and Language, volume 66(4), pages 789–806, 2012. Elsevier. doi: 10.1016/j.jml.2011.11.006

[51] de Jong, Gea, Hudson, Toby, Nolan, Francis, McDougall, Kirsty. The telephone effect on F0. IAFPA 2011 conference, Vienna, Austria, volume 11, 2011.

